# Crystal structure of the new hybrid material bis­(1,4-diazo­niabi­cyclo­[2.2.2]octa­ne) di-μ-chlorido-bis­[tetra­chlorido­bis­muthate(III)] dihydrate

**DOI:** 10.1107/S2056989015019933

**Published:** 2015-10-28

**Authors:** Marwen Chouri, Habib Boughzala

**Affiliations:** a Laboratoire de Matériaux et Cristallochimie, Faculté des Sciences de Tunis, Université de Tunis El Manar, 2092 Manar II Tunis, Tunisia

**Keywords:** crystal structure, hybrid material, DABCO

## Abstract

The title compound bis­(1,4-diazo­niabi­cyclo­[2.2.2]octa­ne) di-μ-chlorido-bis­[tetra­chlorido­bis­muthate(III)] dihydrate, (C_6_H_14_N_2_)_2_[Bi_2_Cl_10_]·2H_2_O, was ob­tain­ed by slow evaporation at room temperature of a hydro­chloric aqueous solution (pH = 1) containing bis­muth(III) nitrate and 1,4-di­aza­bicyclo­[2.2.2]octane (DABCO) in a 1:2 molar ratio. The structure displays a two-dimensional arrangement parallel to (100) of isolated [Bi_2_Cl_10_]^4−^ bi­octa­hedra (site symmetry -1) separated by layers of organic 1,4-diazo­niabi­cyclo­[2.2.2]octane dications [(DABCOH_2_)^2+^] and water mol­ecules. O—H⋯Cl, N—H⋯O and N—H⋯Cl hydrogen bonds lead to additional cohesion of the structure.

## Abstract 2

The title salt, (C_6_H_14_N_2_)_2_[Bi_2_Cl_10_]·2H_2_O, bears a close resemblance to its homologous anti­monate structure. The crystal structure is formed by an alternating packing of organic and inorganic layers along [001] and contains isolated (Bi_2_Cl_10_)^4−^ bi­octa­hedra.

## Chemical context   

In recent years, many new organic–inorganic hybrid com­pounds have been synthesized because of their inter­esting physical behaviour and applications in optoelectronics (Jakubas & Sobczyk, 1990 ▸). The main inter­esting optical activity observed in this kind of compounds is generally the result of the presence of an active *ns* ^2^ lone pair (Chaabouni *et al.*, 1998 ▸) in the inorganic parts. It can also be the result of an important structural distortion in the organic cations (Ishihara *et al.*, 1990 ▸; Lacroix *et al.*, 1994 ▸). The combination of the particular properties of the organic and inorganic moieties can induce inter­esting new properties. In particular for the halogenated bis­muth or anti­mony anionic networks (Ahmed *et al.*, 2001 ▸; Jakubas *et al.*, 2005 ▸), the anionic arrangement leads to four kinds of dimensionalities: quantum dots (zero-di­men­sional, 0D) observed in hybrids such as (C_6_H_14_N_2_)_2_[Sb_2_Cl_10_]·2H_2_O (Ben Rhaiem *et al.*, 2013 ▸), quantum wires (one-dimensional, 1D) as is the case in the structure of (C_2_H_7_N_4_O)_2_ [BiCl_5_] (Ferjani *et al.*, 2012 ▸), quantum wells (two-dimensional, 2D) and a bulk (three-dimensional, 3D) topology. The organic cations are usually filling the empty space left by the inorganic network. Here we report the structure of a new hybrid bis­muthate compounds having a 0D dimensionality with respect to its inorganic part.
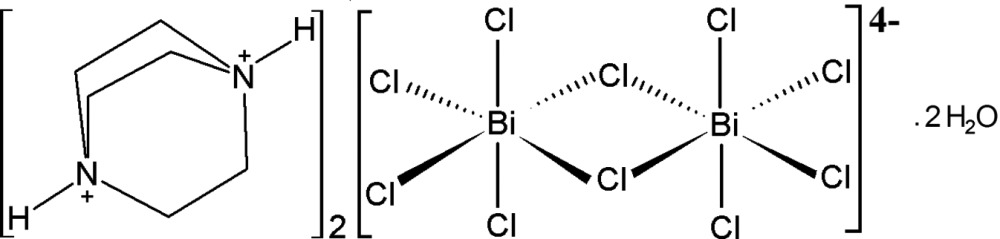


## Structural commentary   

**Figure 1:**
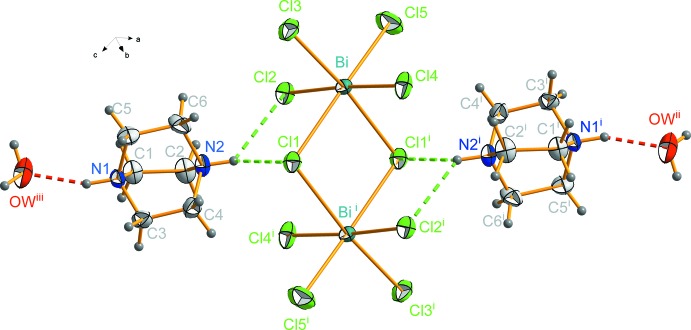
Plot of the mol­ecular entities of (C_6_H_14_N_2_)_2_[Bi_2_Cl_10_]. 2H_2_O, showing the atom numbering scheme. Atomic displacement ellipsoids are drawn at the 50% probability level and H atoms are shown as small spheres of arbitrary radius. [Symmetry codes: (i) −*x* + 1, −*y* + 1, −*z* + 1; (ii) −*x* + 2, *y* + 0.5, −*z* + 0.5; (iii) *x* − 1, −*y* + 0.5, *z* + 0.5.]

The structural unit (Fig. 1 ▸) of the compound is built up by an isolated dimeric deca­chlorido­bis­muthate(III) [Bi_2_Cl_10_]^4−^ anion, two organic 1,4-diazo­niabi­cyclo­[2.2.2]octane dications [(DABCOH_2_)^2+^] and two water mol­ecules. These components are linked by strong hydrogen bonds. The inorganic moiety is an edge-sharing di­octa­hedron located site with symmetry 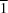. The two (DABCOH_2_)^2+^ dications (Fig. 4 ▸) in the structural unit are related to the dimeric [Bi_2_Cl_10_]^4−^ units by means of N2—H2⋯Cl2 and N2—H2⋯Cl1 inter­actions.

The bond lengths and angles of the dication are within normal ranges and are comparable to those observed in similar structures. Table 1 ▸ summarizes the most important distances in these mol­ecules. The C—N bond lengths vary from 1.479 (11) to 1.508 (12) Å. The C—C bond lengths vary from 1.500 (13) to 1.535 (13) Å. The angles in this mol­ecule are between 109.8 (7) and 110.7 (8)° for C—N—C and between 108.1 (8) and 109.2 (8)° for N—C—C.

As listed in Table 1 ▸, the bond lengths of bis­muth to terminal chlorides [2.587 (5)–2.704 (5) Å] are shorter than the bridging ones [2.863 (4) and 2.884 (4) Å]. The Cl—Bi—Cl angles vary from 84.46 (12) to 95.4 (2)° for the *cis* and 173.25 (15) to 176.64 (15)° for the *trans* arrangement. Using Shannon’s method (Shannon, 1976 ▸), the distortion index of 1.87 (9) × 10^−3^ reveals only a small distortion in the BiCl_6_ octa­hedron. The bis­muth 6*s* ^2^ electron pair has stereochemical activity and the hydrogen-bond orientation can be related to the bis­muth polyhedra distortion. The final Fourier difference map reveals four large peaks at approximately 1 Å from the bis­muth atom that can be attributed to the delocalization of the 6*s* ^2^ electron pair as is the case in most other bis­muth-based structures.

The (C_6_H_14_N_2_)_2_[Bi_2_Cl_10_]·2H_2_O structure is very close to that of (C_6_H_14_N_2_)_2_[Sb_2_Cl_10_]·2H_2_O (Ben Rhaiem *et al.*, 2013 ▸). The cell parameters of both structures can be compared after making a necessary transformation (*cba*) in the *Pnnm* anti­mony unit cell to be comparable to the bis­muth one (Table 2 ▸). Apart from the higher symmetry of the anti­mony structure, an important distortion is noted in the SbCl_6_ octa­hedra confirmed by the Shannon’s distortion index (Shannon,1976 ▸) [6.20 (9) × 10^−3^], more than three times larger than the one for the title bis­muth compound [1.87 (9) × 10^−3^] . It is worth noting that the water mol­ecule plays a more efficient role in the bis­muth based compound. In (C_6_H_14_N_2_)_2_[Sb_2_Cl_10_]·2H_2_O, the H_2_O mol­ecules are only linked to (DABCOH_2_)^2+^ and in the (C_6_H_14_N_2_)_2_[Bi_2_Cl_10_]·2H_2_O structure they are directly hydrogen bonded to both the organic and inorganic parts (Fig. 3 ▸). The atomic radius of bis­muth is larger than that for anti­mony, and thus an increase of the cell volume is expected. In fact, the main increase is observed for the *c* axis [13.99 (2) Å] because the metallic coordination polyhedra are aligned along this axis. On the other hand, a roughly equivalent decrease of the *b* parameter is observed causing the unit-cell volume of the two compounds approximately to be the same. A general comparison of the two structures reveals that they have a similar 3D pattern, built up by isolated bi­octa­hedra, (DABCOH_2_)^2+^ cations and water mol­ecules leaving empty the same voids. On the other hand, the water mol­ecule immediate environment is more regular in the Sb structure (Fig. 3 ▸ *b*) and the (DABCOH_2_)^2+^ cation is more distorted in the Bi structure (Fig. 3 ▸ *a*) explaining the lowering of the symmetry in the title compound.

## Supra­molecular features   

**Figure 2:**
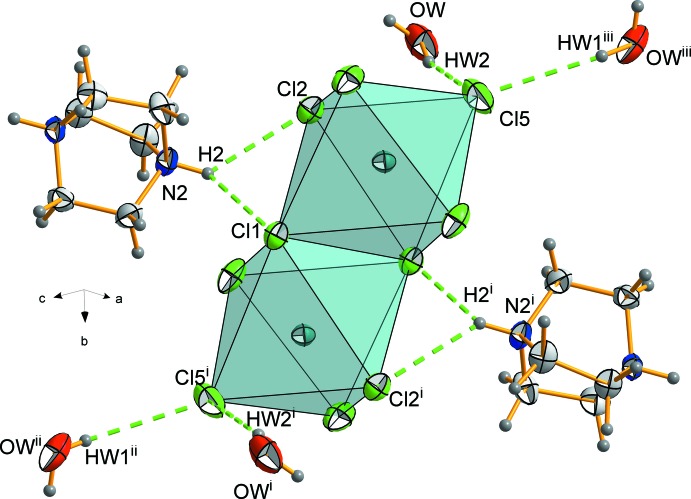
Hydrogen-bonding environment of the anionic part of the structure. [Symmetry codes: (i) −*x* + 1, −*y* + 1, −*z* + 1; (ii) −*x* + 1, −*y* + 1, −*z* + 1.5; (iii) *x*, −*y* + 0.5, *z* − 0.5.]

**Figure 3:**
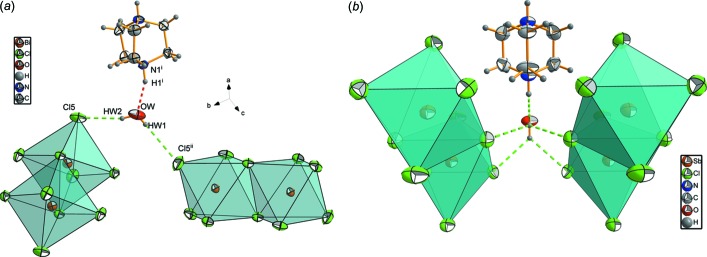
Water-mol­ecule hydrogen-bonding inter­action between organic and inorganic parts: (*a*) in the title compound [symmetry codes: (i) *x* + 1, −*y* + 0.5, *z* − 0.5; (ii) *x*, −*y* + 0.5, *z* + 0.5]; (*b*) in the structure of (C_6_H_14_N_2_)_2_[Sb_2_Cl_10_]·2H_2_O

**Figure 4:**
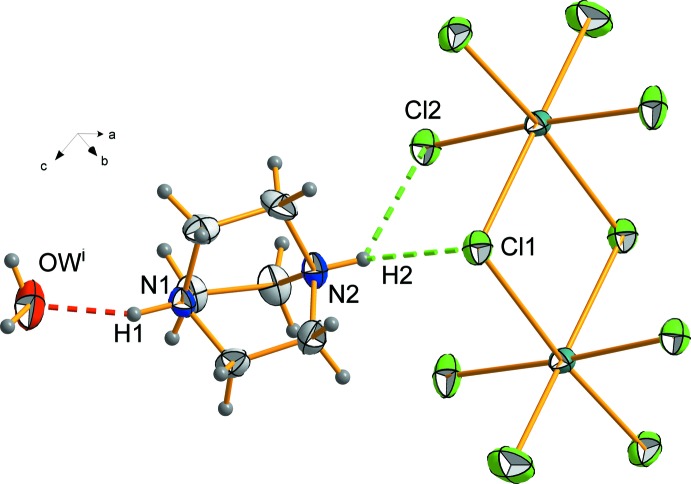
Hydrogen-bonding environment of the cationic organic part of the title compound.

**Figure 5:**
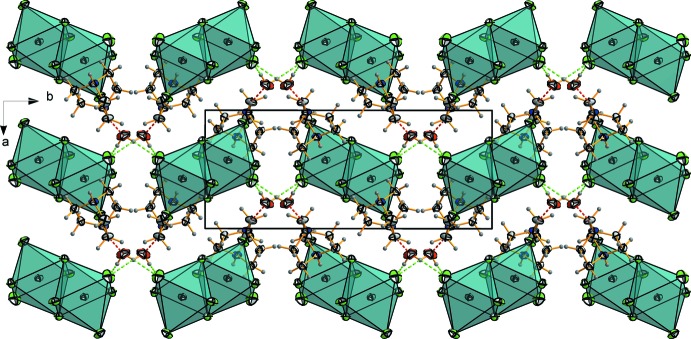
Projection of the crystal structure of the bis­muthate hybrid compound along the *c* axis, showing the alternation of organic and inorganic layers.

As shown in Fig. 2 ▸, every anionic unit is linked to four water mol­ecules and two organic cations. The water mol­ecules (Fig. 3 ▸) are strongly hydrogen bonded to the inorganic part by means of O—H*W*1⋯Cl5^ii^ [symmetry code: (ii) *x*, −*y* + 0.5, *z* + 0.5] and O—H*W*2⋯Cl5 inter­actions. The DABCO cations are hydrogen bonded to water mol­ecules, leading to chains composed of organic moieties, inorganic clusters and H_2_O mol­ecules running along the *b* direction (Fig. 1 ▸). The water mol­ecules stabilize the structure by playing a bridge role between organic and inorganic parts. Furthermore, they ensure the link in the other directions leading to a hydrogen-bond-based three-dimensional network. The structure can be seen (Fig. 5 ▸) as an alternation of organic and inorganic layers parallel to (100) which are linked by a strong hydrogen-bond pattern (Table 3 ▸).

## Synthesis and crystallization   

(C_6_H_14_N_2_)_2_[Bi_2_Cl_10_]·2H_2_O crystals were obtained at ambient conditions by dissolving Bi(NO_3_)_3_·5H_2_O and DABCO (C_6_H_12_N_2_) in water in a 1:2 molar ratio. The pH of the solution was adjusted to 1 with HCl. The mixture was stirred and kept for several days. Colourless crystals were obtained after a few weeks.

## Refinement   

Crystal data, data collection and structure refinement details are summarized in Table 4 ▸. The isotropic displacement parameter of the hydrogen atoms for the water mol­ecule were fixed to be restrained to be approximately 1.5 times those of the parent atom and the water mol­ecule geometries were regularised using distance restraints

## Supplementary Material

Crystal structure: contains datablock(s) I. DOI: 10.1107/S2056989015019933/vn2102sup1.cif

Structure factors: contains datablock(s) I. DOI: 10.1107/S2056989015019933/vn2102Isup2.hkl

CCDC reference: 971956

Additional supporting information:  crystallographic information; 3D view; checkCIF report

## Figures and Tables

**Table 1:** Selected geometric parameters ()

BiCl5	2.588(2)	N1C5	1.504(9)
BiCl3	2.601(2)	N2C4	1.489(10)
BiCl2	2.6611(19)	N2C2	1.492(9)
BiCl4	2.704(2)	N2C6	1.494(10)
BiCl1	2.8610(19)	C1C2	1.517(11)
BiCl1^i^	2.884(2)	C6C5	1.531(11)
N1C1	1.485(9)	C3C4	1.493(11)
N1C3	1.503(9)		

Symmetry code: (i) 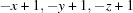.

**Table 2:** Comparison of the cell parameters of the structures of [Bi_2_Cl_10_](C_6_H_14_N_2_)_2_2H_2_O and [Sb_2_Cl_10_](C_6_H_14_N_2_)_2_2H_2_O.

	[Bi_2_Cl_10_](C_6_H_14_N_2_)_2_2H_2_O	[Sb_2_Cl_10_](C_6_H_14_N_2_)_2_2H_2_O	Parameter variation (%) [(*X*_Bi_ *X*_Sb_)/(*X*_Sb_)]100
Crystal system	monoclinic	orthorhombic	-
Space group	*P*2_1_/*c*	*Pnnm* *Pnmn* (cba)	-
*a* ()	7.875(3)	9.162(1) => 7.566(2)	4.08(2)
*b* ()	18.379(5)	20.689(7) => 20.689(7)	11.16(3)
*c* ()	10.444(4)	7.566(2) => 9.162(1)	13.99(2)
()	105.95(3)	90.00	-
*V* (^3^)	1453.4(9)	1446.8(7)	0.45(7)

**Table 3:** Hydrogen-bond geometry (, )

*D*H*A*	*D*H	H*A*	*D* *A*	*D*H*A*
OwHw2Cl5	0.91	2.63	3.458(8)	163
N1H1Ow^ii^	0.91	1.87	2.739(10)	159
OwHw1Cl5^iii^	0.91	2.80	3.475(9)	138
N2H2Cl1	0.91	2.73	3.352(6)	127
N2H2Cl2	0.91	2.65	3.325(7)	132

Symmetry codes: (ii) 
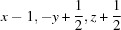
; (iii) 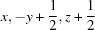.

**Table 4:** Experimental details

Crystal data	
Chemical formula	(C_6_H_14_N_2_)_2_[Bi_2_Cl_10_]2H_2_O
*M*_r_	1036.88
Crystal system, space group	Monoclinic, *P*2_1_/*c*
Temperature (K)	293
*a*, *b*, *c* ()	7.875(3), 18.379(5), 10.444(4)
()	105.95(3)
*V* (^3^)	1453.4(9)
*Z*	2
Radiation type	Mo *K*
(mm^1^)	13.03
Crystal size (mm)	0.5 0.3 0.2

Data collection	
Diffractometer	EnrafNonius CAD-4
Absorption correction	scan (North *et al.*, 1968 ▸)
*T*_min_, *T*_max_	0.013, 0.074
No. of measured, independent and observed [*I* > 2(*I*)] reflections	3159, 3159, 2681
*R*_int_	0.035
(sin /)_max_ (^1^)	0.638

Refinement	
*R*[*F* ^2^ > 2(*F* ^2^)], *wR*(*F* ^2^), *S*	0.036, 0.102, 1.06
No. of reflections	3159
No. of parameters	142
No. of restraints	2
H-atom treatment	H atoms treated by a mixture of independent and constrained refinement
_max_, _min_ (e ^3^)	3.48, 2.57

Computer programs: *CAD-4 EXPRESS* (EnrafNonius, 1994 ▸), *XCAD4* (Harms Wocadlo, 1995 ▸), *SHELXS97* (Sheldrick, 2008 ▸), *SHELXL2014* (Sheldrick, 2015 ▸), *DIAMOND* (Brandenburg, 2006 ▸) and *publCIF* (Westrip, 2010 ▸).

## supplementary crystallographic information

### Crystal data

**Table:**

(C_6_H_14_N_2_)_2_[Bi_2_Cl_10_]·2H_2_O	*F*(000) = 968
*M**_r_* = 1036.88	*D*_x_ = 2.369 Mg m^−^^3^
Monoclinic, *P*2_1_/*c*	Mo *K*α radiation, λ = 0.71073 Å
*a* = 7.875 (3) Å	Cell parameters from 3158 reflections
*b* = 18.379 (5) Å	θ = 2.2–2.7°
*c* = 10.444 (4) Å	µ = 13.03 mm^−^^1^
β = 105.95 (3)°	*T* = 293 K
*V* = 1453.4 (9) Å^3^	Prism, colourless
*Z* = 2	0.5 × 0.3 × 0.2 mm

### Data collection

**Table:**

Enraf–Nonius CAD-4 diffractometer	2681 reflections with *I* > 2σ(*I*)
Radiation source: fine-focus sealed tube	*R*_int_ = 0.035
Graphite monochromator	θ_max_ = 27.0°, θ_min_ = 2.2°
ω/2θ scans	*h* = −10→1
Absorption correction: ψ scan North *et al.* (1968). Number of ψ scan sets used was 5 Theta correction was applied. Averaged transmission function was used. No Fourier smoothing was applied.	*k* = −1→23
*T*_min_ = 0.013, *T*_max_ = 0.074	*l* = −12→13
3159 measured reflections	2 standard reflections every 120 min
3159 independent reflections	intensity decay: 10%

### Refinement

**Table:**

Refinement on *F*^2^	2 restraints
Least-squares matrix: full	Hydrogen site location: mixed
*R*[*F*^2^ > 2σ(*F*^2^)] = 0.036	H atoms treated by a mixture of independent and constrained refinement
*wR*(*F*^2^) = 0.102	*w* = 1/[σ^2^(*F*_o_^2^) + (0.0625*P*)^2^ + 4.4831*P*] where *P* = (*F*_o_^2^ + 2*F*_c_^2^)/3
*S* = 1.06	(Δ/σ)_max_ < 0.001
3159 reflections	Δρ_max_ = 3.48 e Å^−^^3^
142 parameters	Δρ_min_ = −2.57 e Å^−^^3^

### Special details

**Table:**

Geometry. All e.s.d.'s (except the e.s.d. in the dihedral angle between two l.s. planes) are estimated using the full covariance matrix. The cell e.s.d.'s are taken into account individually in the estimation of e.s.d.'s in distances, angles and torsion angles; correlations between e.s.d.'s in cell parameters are only used when they are defined by crystal symmetry. An approximate (isotropic) treatment of cell e.s.d.'s is used for estimating e.s.d.'s involving l.s. planes.

### Fractional atomic coordinates and isotropic or equivalent isotropic displacement parameters (Å^2^)

**Table:**

	*x*	*y*	*z*	*U*_iso_*/*U*_eq_	
Bi	0.42097 (3)	0.42073 (2)	0.34670 (2)	0.02518 (11)	
Cl1	0.2576 (2)	0.50934 (10)	0.50438 (18)	0.0329 (4)	
Cl2	0.4635 (3)	0.32483 (10)	0.54389 (18)	0.0361 (4)	
Cl3	0.1117 (3)	0.36755 (11)	0.2279 (2)	0.0441 (5)	
Cl4	0.3821 (3)	0.52442 (12)	0.1571 (2)	0.0438 (5)	
Cl5	0.5941 (4)	0.33648 (14)	0.2288 (3)	0.0635 (7)	
OW	0.7852 (9)	0.2151 (4)	0.4798 (8)	0.0609 (18)	
HW1	0.701 (11)	0.194 (6)	0.501 (12)	0.070*	
HW2	0.732 (14)	0.251 (4)	0.434 (10)	0.070*	
N1	0.0157 (8)	0.3538 (3)	0.8660 (6)	0.0291 (12)	
H1	−0.0668	0.3405	0.9173	0.035*	
N2	0.2250 (8)	0.3886 (3)	0.7369 (6)	0.0319 (13)	
H2	0.3082	0.4026	0.6866	0.038*	
C1	0.1780 (10)	0.3089 (4)	0.9111 (8)	0.0340 (16)	
H1A	0.1513	0.2584	0.8867	0.041*	
H1B	0.2238	0.3117	1.0072	0.041*	
C6	0.0703 (11)	0.3511 (5)	0.6456 (7)	0.0403 (19)	
H6A	0.1062	0.3045	0.6179	0.048*	
H6B	0.0224	0.3805	0.5668	0.048*	
C3	0.0613 (10)	0.4331 (4)	0.8868 (8)	0.0344 (16)	
H3A	−0.0457	0.4618	0.8708	0.041*	
H3B	0.1310	0.4413	0.9778	0.041*	
C4	0.1641 (11)	0.4551 (4)	0.7924 (8)	0.0385 (17)	
H4A	0.0904	0.4841	0.7209	0.046*	
H4B	0.2650	0.4843	0.8386	0.046*	
C5	−0.0695 (11)	0.3398 (5)	0.7206 (7)	0.0386 (17)	
H5A	−0.1673	0.3731	0.6874	0.046*	
H5B	−0.1147	0.2905	0.7078	0.046*	
C2	0.3144 (11)	0.3374 (4)	0.8454 (8)	0.0385 (17)	
H2A	0.4078	0.3625	0.9105	0.046*	
H2B	0.3663	0.2973	0.8090	0.046*	

### Atomic displacement parameters (Å^2^)

**Table:**

	*U*^11^	*U*^22^	*U*^33^	*U*^12^	*U*^13^	*U*^23^
Bi	0.02361 (17)	0.02675 (16)	0.02414 (16)	−0.00230 (9)	0.00482 (11)	−0.00228 (8)
Cl1	0.0253 (8)	0.0374 (9)	0.0354 (9)	0.0006 (7)	0.0073 (7)	0.0026 (7)
Cl2	0.0343 (9)	0.0360 (9)	0.0374 (9)	0.0016 (7)	0.0087 (7)	0.0066 (7)
Cl3	0.0352 (10)	0.0522 (11)	0.0396 (10)	−0.0140 (9)	0.0011 (8)	0.0003 (8)
Cl4	0.0282 (9)	0.0570 (12)	0.0434 (10)	−0.0072 (8)	0.0052 (8)	0.0165 (9)
Cl5	0.0703 (16)	0.0640 (15)	0.0643 (15)	0.0110 (12)	0.0319 (13)	−0.0197 (12)
OW	0.045 (4)	0.073 (5)	0.067 (5)	0.017 (3)	0.019 (3)	−0.016 (4)
N1	0.024 (3)	0.037 (3)	0.030 (3)	−0.001 (2)	0.013 (2)	−0.002 (2)
N2	0.027 (3)	0.035 (3)	0.037 (3)	0.003 (2)	0.016 (3)	0.005 (3)
C1	0.032 (4)	0.034 (4)	0.036 (4)	0.010 (3)	0.009 (3)	0.011 (3)
C6	0.044 (5)	0.054 (5)	0.021 (3)	0.002 (4)	0.005 (3)	−0.003 (3)
C3	0.032 (4)	0.030 (3)	0.043 (4)	0.002 (3)	0.012 (3)	−0.007 (3)
C4	0.038 (4)	0.030 (4)	0.050 (5)	−0.003 (3)	0.017 (4)	0.001 (3)
C5	0.033 (4)	0.050 (4)	0.028 (4)	−0.005 (3)	−0.001 (3)	−0.005 (3)
C2	0.034 (4)	0.041 (4)	0.040 (4)	0.012 (3)	0.010 (3)	0.014 (3)

### Geometric parameters (Å, º)

**Table:**

Bi—Cl5	2.588 (2)	N2—H2	0.9800
Bi—Cl3	2.601 (2)	C1—C2	1.517 (11)
Bi—Cl2	2.6611 (19)	C1—H1A	0.9700
Bi—Cl4	2.704 (2)	C1—H1B	0.9700
Bi—Cl1	2.8610 (19)	C6—C5	1.531 (11)
Bi—Cl1^i^	2.884 (2)	C6—H6A	0.9700
Cl1—Bi^i^	2.884 (2)	C6—H6B	0.9700
OW—HW1	0.850 (10)	C3—C4	1.493 (11)
OW—HW2	0.850 (10)	C3—H3A	0.9700
N1—C1	1.485 (9)	C3—H3B	0.9700
N1—C3	1.503 (9)	C4—H4A	0.9700
N1—C5	1.504 (9)	C4—H4B	0.9700
N1—H1	0.9800	C5—H5A	0.9700
N2—C4	1.489 (10)	C5—H5B	0.9700
N2—C2	1.492 (9)	C2—H2A	0.9700
N2—C6	1.494 (10)	C2—H2B	0.9700
			
Cl5—Bi—Cl3	95.45 (9)	C2—C1—H1B	110.0
Cl5—Bi—Cl2	90.07 (8)	H1A—C1—H1B	108.4
Cl3—Bi—Cl2	91.28 (7)	N2—C6—C5	108.1 (6)
Cl5—Bi—Cl4	92.39 (9)	N2—C6—H6A	110.1
Cl3—Bi—Cl4	90.73 (7)	C5—C6—H6A	110.1
Cl2—Bi—Cl4	176.66 (6)	N2—C6—H6B	110.1
Cl5—Bi—Cl1	173.58 (7)	C5—C6—H6B	110.1
Cl3—Bi—Cl1	88.74 (7)	H6A—C6—H6B	108.4
Cl2—Bi—Cl1	84.97 (6)	C4—C3—N1	108.6 (6)
Cl4—Bi—Cl1	92.41 (7)	C4—C3—H3A	110.0
Cl5—Bi—Cl1^i^	91.38 (8)	N1—C3—H3A	110.0
Cl3—Bi—Cl1^i^	173.16 (6)	C4—C3—H3B	110.0
Cl2—Bi—Cl1^i^	88.44 (6)	N1—C3—H3B	110.0
Cl4—Bi—Cl1^i^	89.25 (6)	H3A—C3—H3B	108.4
Cl1—Bi—Cl1^i^	84.43 (6)	N2—C4—C3	109.0 (6)
Bi—Cl1—Bi^i^	95.57 (6)	N2—C4—H4A	109.9
HW1—OW—HW2	102 (10)	C3—C4—H4A	109.9
C1—N1—C3	110.0 (6)	N2—C4—H4B	109.9
C1—N1—C5	109.4 (6)	C3—C4—H4B	109.9
C3—N1—C5	109.5 (6)	H4A—C4—H4B	108.3
C1—N1—H1	109.3	N1—C5—C6	108.0 (6)
C3—N1—H1	109.3	N1—C5—H5A	110.1
C5—N1—H1	109.3	C6—C5—H5A	110.1
C4—N2—C2	110.9 (6)	N1—C5—H5B	110.1
C4—N2—C6	109.5 (6)	C6—C5—H5B	110.1
C2—N2—C6	109.1 (6)	H5A—C5—H5B	108.4
C4—N2—H2	109.1	N2—C2—C1	108.5 (6)
C2—N2—H2	109.1	N2—C2—H2A	110.0
C6—N2—H2	109.1	C1—C2—H2A	110.0
N1—C1—C2	108.6 (5)	N2—C2—H2B	110.0
N1—C1—H1A	110.0	C1—C2—H2B	110.0
C2—C1—H1A	110.0	H2A—C2—H2B	108.4
N1—C1—H1B	110.0		

Symmetry code: (i) −*x*+1, −*y*+1, −*z*+1.

### Hydrogen-bond geometry (Å, º)

**Table:**

*D*—H···*A*	*D*—H	H···*A*	*D*···*A*	*D*—H···*A*
Ow—Hw2···Cl5	0.91	2.63	3.458 (8)	163
N1—H1···Ow^ii^	0.91	1.87	2.739 (10)	159
Ow—Hw1···Cl5^iii^	0.91	2.80	3.475 (9)	138
N2—H2···Cl1	0.91	2.73	3.352 (6)	127
N2—H2···Cl2	0.91	2.65	3.325 (7)	132

Symmetry codes: (ii) *x*−1, −*y*+1/2, *z*+1/2; (iii) *x*, −*y*+1/2, *z*+1/2.
